# [Retracted] MEG3/miR‑21 axis affects cell mobility by suppressing epithelial‑mesenchymal transition in gastric cancer

**DOI:** 10.3892/or.2024.8799

**Published:** 2024-08-19

**Authors:** Gang Xu, Lei Meng, Dawei Yuan, Kang Li, Yong Zhang, Chengxue Dang, Kun Zhu

Oncol Rep 40: 39–48, 2018; DOI: 10.3892/or.2018.6424

Following the publication of this paper, it was drawn to the Editor's attention by a concerned reader that certain of the Transwell cell invasion assay data shown in Fig. 2B on p. 42 and the immunofluorescence data shown in Fig. 4D on p. 44 were strikingly similar to data appearing in other articles written by different authors at different research institutes that were submitted to different journals at around the same time. Moreover, a further investigation of this paper undertaken by the Editorial Office identified a large number of overlapping data panels comparing the Transwell cell migration and invasion assay data and the scratch-wound assay data both within and between Figs. 2 and 3, where data which were intended to have shown the results from differently performed experiments had apparently been derived from the same original source, including an overlapping section of data within the ‘MEG3+mimic’ panel in Fig. 3G that would be difficult to attribute to pure chance. Owing to the fact that the contentious data in the above article had already been submitted for publication at around the same time as its submission to *Oncology Reports*, and given an overall lack of confidence in the presented data, the Editor has decided that this paper should be retracted from the Journal. The authors were asked for an explanation to account for these concerns, but the Editorial Office did not receive a reply. The Editor apologizes to the readership for any inconvenience caused.

